# Association between *ACE2* and *TMPRSS2* nasopharyngeal expression and COVID-19 respiratory distress

**DOI:** 10.1038/s41598-021-88944-8

**Published:** 2021-05-06

**Authors:** Átila Duque Rossi, João Locke Ferreira de Araújo, Tailah Bernardo de Almeida, Marcelo Ribeiro-Alves, Camila de Almeida Velozo, Jéssica Maciel de Almeida, Isabela de Carvalho Leitão, Sâmila Natiane Ferreira, Jéssica da Silva Oliveira, Hugo José Alves, Helena Toledo Scheid, Débora Souza Faffe, Rafael Mello Galliez, Renata Eliane de Ávila, Gustavo Gomes Resende, Mauro Martins Teixeira, COVID-19 UFRJ Workgroup, Alice Laschuk Herlinger, Aliny dos Santos Carvalho, André Felipe Andrade dos Santos, Anna Carla Pinto Castiñeiras, Bianca Isabelle Barreto Teixeira, Bianca Ortiz da Silva, Bruno Clarkson, Bruno Eduardo Dematté, Camila Nacif, Camille Victória Leal Correia de Silva, Carolina Moreira Voloch, Caroline Macedo Nascimento, Carolyne Lalucha Alves L. da Graça, Cassia Cristina Alves Gonçalves, Cíntia Policarpo, Diana Mariani, Ekaterini Simões Goudouri, Elaine Sobral da Costa, Elisangela Costa da Silva, Enrico Bruno Riscarolli, Érica Ramos dos Santos Nascimento, Fabio Hecht Castro Medeiros, Fábio Luís Lima Monteiro, Fernanda Leitão dos Santos, Fernando Luz de Castro, Filipe Romero Rebello Moreira, Francine Bittencourt Schiffler, Gabriela Bergiante Kraychete, Gabriele Silveira da Cunha, Gisely Novaes Borges da Cunha, Guilherme Sant’Anna de Lira, Gustavo Peixoto Duarte da Silva, Harrison James Westgarth, Helena D.’Anunciação de Oliveira, Helena Keito Toma, Huang Ling Fang, Inês Corrêa Gonçalves, Ingrid Camelo da Silva, Isabela Labarba Carvalho de Almeida, Joissy Aprigio de Oliveira, Juliana Cazarin de Menezes, Juliana Tiemi Sato Fortuna, Karyne Ferreira Monteiro, Kissyla Harley Della Pascoa França, Laura Zalcberg Renault, Lendel Correia da Costa, Leticia Averbug Correa, Liane de Jesus Ribeiro, Lídia Theodoro Boullosa, Liliane Tavares de Faria Cavalcante, Luana dos Santos Costa, Lucas Matos Millioni, Luciana Jesus da Costa, Luiza Mendonça Higa, Marcela dos Santos Durães, Marcelo Amaral de Souza, Marcelo Calado de Paula Tôrres, Mariana Freire Campos, Mariana Quinto, Mariane Talon de Menezes, Marisa Souza Correia, Mateus Rodrigues de Queiroz, Matheus Augusto Calvano Cosentino, Mayla Gabryele Miranda de Melo, Mirela D’arc Ferreira da Costa, Pedro Henrique Costa da Paz, Raissa Mirella dos Santos Cunha da Costa, Raquel Fernandes Coelho, Richard Araujo Maia, Rodrigo de Moraes Brindeiro, Romina Carvalho Ferreira, Sérgio Machado Lisboa, Thamiris dos Santos Miranda, Victor Akira Ota, Victoria Cortes Bastos, Viviane Guimarães Gomes, Orlando da Costa Ferreira Júnior, Terezinha Marta P. P. Castiñeiras, Renan Pedra Souza, Amilcar Tanuri, Renato Santana de Aguiar, Shana Priscila Coutinho Barroso, Cynthia Chester Cardoso

**Affiliations:** 1 Laboratório de Virologia Molecular, Departamento de Genética, Instituto de Biologia, Universidade Federal do Rio de Janeiro, Av. Carlos Chagas Filho 373, CCS, Bloco A, Sala 121 Ilha do Fundão, Rio de Janeiro, RJ 21941-902 Brazil; 2 Laboratório de Biologia Integrativa, Departamento de Genética, Ecologia e Evolução, Instituto de Ciências Biológicas, Universidade Federal de Minas Gerais, Belo Horizonte, 31270-901 Brazil; 3 Instituto de Estudos do Mar Almirante Paulo Moreira, Marinha do Brasil, Arraial do Cabo, 28930-000 Brazil; 4 Instituto Nacional de Infectologia Evandro Chagas, Fiocruz, Rio de Janeiro, 21040-360 Brazil; 5 Instituto de Biofísica, Universidade Federal do Rio de Janeiro, Rio de Janeiro, 21941-902 Brazil; 6 Instituto de Pesquisas Biomédicas, Hospital Naval Marcílio Dias, Marinha do Brasil, Rio de Janeiro, 20725-090 Brazil; 7 Faculdade de Medicina, Universidade Federal do Rio de Janeiro, Rio de Janeiro, 21941-902 Brazil; 8 Hospital Eduardo de Menezes, Belo Horizonte, 30622-020 Brazil; 9 Hospital das Clínicas, Universidade Federal de Minas Gerais (HC-UFMG/EBSERH), Belo Horizonte, 30130-100 Brazil; 10 Departamento de Bioquímica e Imunologia, Instituto de Ciências Biológicas, Universidade Federal de Minas Gerais, Belo Horizonte, 31270-901 Brazil; 11 Centro de Triagem e Diagnóstico COVID-19 UFRJ, Universidade Federal do Rio de Janeiro, Rio de Janeiro, 21941-902 Brazil

**Keywords:** Virology, SARS-CoV-2, Viral infection, Gene expression, Infectious diseases, Respiratory tract diseases, Epidemiology, Transcription

## Abstract

ACE2 and TMPRSS2 are key players on SARS-CoV-2 entry into host cells. However, it is still unclear whether expression levels of these factors could reflect disease severity. Here, a case–control study was conducted with 213 SARS-CoV-2 positive individuals where cases were defined as COVID-19 patients with respiratory distress requiring oxygen support (N = 38) and controls were those with mild to moderate symptoms of the disease who did not need oxygen therapy along the entire clinical course (N = 175). *ACE2* and *TMPRSS2* mRNA levels were evaluated in nasopharyngeal swab samples by RT-qPCR and logistic regression analyzes were applied to estimate associations with respiratory outcomes. *ACE2* and *TMPRSS2* levels positively correlated with age, which was also strongly associated with respiratory distress. Increased nasopharyngeal *ACE2* levels showed a protective effect against this outcome (_adj_OR = 0.30; 95% CI 0.09–0.91), while TMPRSS2/ACE2 ratio was associated with risk (_adj_OR = 4.28; 95% CI 1.36–13.48). On stepwise regression, TMPRSS2/ACE2 ratio outperformed *ACE2* to model COVID-19 severity. When nasopharyngeal swabs were compared to bronchoalveolar lavages in an independent cohort of COVID-19 patients under mechanical ventilation, similar expression levels of these genes were observed. These data suggest nasopharyngeal TMPRSS2/ACE2 as a promising candidate for further prediction models on COVID-19.

## Introduction

In late December 2019, SARS-CoV-2 has emerged as the etiologic agent of a novel human respiratory disease known as coronavirus disease 2019 (COVID-19)^1^. Most COVID-19 patients show mild to moderate symptoms of common respiratory viral infections such as dry cough, headache, and fever. However, some infected individuals may develop intense respiratory distress, pulmonary infiltrates, and secondary bacterial pneumonia^2^. This scenario can evolve to an even more critical condition of respiratory failure, septic shock and multi-organ disfunction^2^. Risk factors such as age, sex (male), hypertension, obesity, diabetes and smoking habit have been identified by their association with hospitalization or death due to COVID-19 on previous epidemiological studies^3–5^.

The angiotensin-converting enzyme 2 (ACE2) has been described as the entry receptor for SARS-CoV-2 and the transmembrane serine protease 2 (TMPRSS2) as an important priming enzyme required during this process^6^. Co-expression of *ACE2* and *TMPRSS2* genes has been detected by single-cell RNA-sequencing analyses on goblet secretory cells (nasal mucosa), type-2 pneumocytes (lungs), and absorptive enterocytes (small intestine), characterizing potential initial target sites for SARS-CoV-2 replication in humans^7^. However, data from *ACE2* and *TMPRSS2* expression on different airway tract sites of SARS-CoV-2 infected individuals is still sparse and have been raising divergent hypothesis about their potential impact on COVID-19 susceptibility.

Recent data found that expression levels of *ACE2* were lower in the nasal epithelium of children under 10 years old, when compared to higher age groups, suggesting that age related *ACE2* expression could help to explain lower susceptibility to SARS-CoV-2 among young people^8^. Moreover, evidence support that higher *ACE2* expression levels could be expected on individuals with hypertension and upon angiotensin receptor blocker therapy^9,10^. Higher levels of *ACE2* have also been found on the respiratory tract of smokers, supporting a risk effect hypothesis for greater *ACE2* expression during SARS-CoV-2 infection^11^. On the other hand, protective effect against acute lung injury/inflammation has already been described for ACE2 and the angiotensin II type 2 receptor (AT2) in mice^12^. ACE2 deficiency has also been linked to exacerbation of adipose tissue inflammation upon high calorie diet induced obesity in mice, reinforcing its potential anti-inflammatory effects^13^.

Thus, the role of *ACE2* expression levels on COVID-19 susceptibility still remains to be determined. Despite the relevance of TMPRSS2 on SARS-CoV-2 entry, poor data is available to understand how its expression is affected by different factors and whether it could be linked to SARS-CoV-2 severity. To further address these questions, here we describe the expression levels of *ACE2* and *TMPRSS2* at the upper respiratory tract from SARS-CoV-2 positive individuals and investigate their association with respiratory distress.

## Methods

### Subjects and study design

A case–control study was carried out including 213 individuals with SARS-CoV-2 infection confirmed by RT-qPCR in nasopharyngeal samples. Patients were enrolled at the Center for COVID-19 diagnosis from the Federal University of Rio de Janeiro (UFRJ) and at Hospital Naval Marcílio Dias (HNMD). The case group was enrolled at HNMD and included 38 individuals that showed intense respiratory distress defined here by their need of oxygen therapy after clinical evaluation (22 received oxygen by nasal cannula, 7 by noninvasive mechanical ventilation and 9 by intubation). The control group included 175 individuals that attended to UFRJ for COVID-19 diagnosis and presented only mild symptoms of COVID-19 along the entire disease course. Frequencies of males and females were paired between cases and controls to avoid confounding.

Clinical information from COVID-19 patients undergoing oxygen therapy (cases) was assessed from medical records and nasopharyngeal swab samples were collected at the time of hospital admission. For mild COVID-19 individuals (controls), information was obtained through a questionnaire application by qualified medical specialists. Exclusion criteria consisted of cancer, autoimmune diseases and/or pregnancy. No smokers were observed among enrolled participants. The present study was performed in accordance with relevant guidelines and regulations and approved by institutional ethical review boards from UFRJ and HNMD (protocol numbers 30161620.0.0000.5257 and 32382820.3.0000.5256 respectively), with written informed consents obtained from all participants.

### Nucleic acid extraction and cDNA synthesis

Total nucleic acid extractions from nasopharyngeal swab samples were performed using the automated *Maxwell System* platform (Promega). 25 μl of total RNA was then submitted to cDNA synthesis with *High-Capacity cDNA Reverse Transcription Kits* (Thermo Fisher Scientific) and stored under − 20 °C.

### Quantitative PCR

Expression levels of *ACE2*, *TMPRSS2,* and *B2M* (as a reference gene) were evaluated by quantitative PCR (qPCR) using *GoTaq Probe qPCR System* (Promega). The specific set of primers and probes (Integrated DNA Technologies) predesigned for exon-exon junctions were used: Hs.PT.58.27645939, Hs.PT.58.39738666 and, Hs.PT.39a.22214847. Reactions were performed in a final volume of 10 µL on a 7500 Real-Time PCR System (Thermo Fisher Scientific) following manufacturer’s recommendations. Genomic DNA amplification was not observed at no-reverse transcriptase controls. For relative quantification of expression levels, the fluorescence accumulation data of real-time RT-PCR reactions of each sample were used for fitting four parameters sigmoid curves (cycle × Rn) to represent each amplification curve using the library qPCR for the R statistical package version 3.0.1. The cycle of quantification (Cq) was determined for each amplification by the maximum of the second derivative of the fitted sigmoid curve. The efficiency of each amplification reaction was calculated as the ratio between the fluorescence (Rn) of the cycle of quantification (Cq) and the fluorescence of the cycle immediately preceding that (Cq-1). For each gene, efficiency was estimated by the mean of all the efficiencies for each amplification reaction for that gene. Once we had the average efficiency of the target (ET) and housekeeping (EH) genes with the quantification cycles (CqT and CqH) for all amplification reactions, we used the following equation to calculate the normalized expression values (Rq) of each target gene for each sample: Rq = ET^ΔCqT^/EH^ΔCqH^^14^. Relative expression results were log-transformed and represented in graphs as mean ± standard error.

### Expression patterns in bronchoalveolar lavage

In addition to the main epidemiological study, an independent case-only cohort of 45 COVID-19 patients under mechanical ventilation was recruited to investigate whether *ACE2* and *TMPRSS2* expression patterns in lower respiratory tract could reflect those observed in the nasopharynx during severe/critical COVID-19. From the 45 samples, 11 were bronchoalveolar lavages (BAL) and 34 were swabs. They were all obtained from distinct individuals of two reference hospitals at the city of Belo Horizonte, also located at Brazilian Southeast region. Nasopharyngeal swabs samples were taken as baseline for comparisons by non-paired statistical analysis. All participants declared written informed consent approved by the institutional ethical review board (CAAE 32224420.3.0000.0008 and CAAE 31462820.3.0000.5149).

### Statistical analyses

In the evaluation of the sociodemographic, clinical, and laboratory features between cases and controls, Wilcoxon rank-sum test were used for continuous numerical variables to test the hypothesis that different groups were drawn from the same distribution or distributions with the same median. Likewise, for categorical nominal variables, Fisher’s exact tests were used to evaluate frequencies among the different groups and test the hypothesis of independence. Kendall’s rank correlation coefficient analyses were estimated for continuous numerical variables. Correlation plots were generated with “ggstatsplot” and “grid” packages. Comparisons of *ACE2* and *TMPRSS2* expression levels between the different groups of interest were performed by contrasts obtained after both bi- and multivariate-linear models fitted by ordinary least square regressions. Normality of continuous data was assessed by Shapiro–Wilk test. A stepwise analysis was conducted to select covariates for multiple logistic regression models using the packages “tidyverse”, “caret”, “leaps” and, “MASS”^15,16^. Association between expression levels and respiratory distress requiring oxygen therapy was performed by crude and adjusted logistic regression models. This model was applied to investigate a role for *ACE2* and *TMPRSS2* expression levels as predictors of respiratory distress (binary outcome). All statistical analyses were performed on R (version 4.0.2).

## Results

### Clinical characteristics of the study population

To investigate whether nasopharyngeal levels of *ACE2* and *TMPRSS2* are associated with respiratory distress susceptibility on COVID-19 patients, 213 SARS-CoV-2 RT-qPCR positive individuals were enrolled in this case–control study. Cases were defined as COVID-19 patients who required oxygen support over the course of SARS-CoV-2 infection while controls were COVID-19 patients that presented only mild symptoms of the disease. The mean age was significantly higher among cases as compared to controls (54 ± 24 *vs* 39 ± 13 years; p = 3.48 × 10^–8^). As expected by the study design, gender distributions were similar between the groups (Table 1). Most common symptoms in the present cohort were cough (70.42%), fever (63.85%), headache (61.50%), anosmia (57.28%) and myalgia (55.87%). Six out of the 13 symptoms evaluated showed significant association with disease severity. Anosmia and headache were significantly more frequent among controls while dyspnea and fatigue were more frequent among cases. All cases used at least one pharmacological intervention against SARS-CoV-2, while about 55% of the control group did so. Six therapeutic strategies (hydroxychloroquine, ceftriaxone, moxifloxacin/ciprofloxacin, levofloxacin, ondansetron, enoxaparin and corticoid use) were found more frequently among cases, though this probably reflects prescription after outcome occurrence (Table 1).

**Table 1:** Epidemiological and demographic description of 213 individuals enrolled on the case–control study.

Variable	Cases		Controls		p-value**
	Total	Mean (SD)	Total	Mean (SD)	
Age	**38**	54 (± 24)	**175**	39 (± 13)	**3.48 × 10**^**–8**^

	Total	N (%)	Total	N (%)	p-value*
Sex	**38**		**175**		0.8435
Female		26 (68.42)		114 (65.14)	
Male		12 (31.58)		61 (34.85)	
Symptoms	**38**		**175**		
Fever		30 (78.95)		106 (60.57)	0.0511
Cough		28 (73.68)		122 (69.71)	0.7718
Sneeze		5 (13.15)		69 (39.42)	0.0038
Dyspnea		31 (81.58)		48 (27.49)	**1.21 × 10**^**–9**^
Coryza/Nasal Congestion		7 (18.42)		69 (39.42)	0.0236
Headache		10 (26.32)		121 (69.14)	**2.21 × 10**^**–6**^
Nausea		4 (10.53)		54 (30.86)	0.0187
Vomit		2 (5.26)		13 (7.43)	0.9019
Diarrhea		6 (15.79)		58 (33.14)	0.0549
Hash		0		11 (07.97)	-
Myalgia		16 (42.11)		103 (58.86)	0.0882
Anosmia		6 (15.79)		116 (66.29)	**3.34 × 10**^**–8**^
Fatigue		10 (26.32)		11 (7.97)	**5.0 × 10**^**–4**^
Treatment	**37**		**149**		
Any Treatment		37 (100)		82 (55.03)	**8.62 × 10**^**–8**^
Dipyrone/Paracetamol		33 (89.19)		120 (80.54)	0.0532
Loratadine		1 (3.03)		5 (3.36)	0.1188
Azithromycin		22 (56.41)		56 (37.58)	0.0051
Amoxicillin/Clavulanate		10 (27.03)		18 (12.08)	0.0079
Hydroxychloroquine		12 (32.43)		6 (4.03)	**4.22 × 10**^**–8**^
Ivermectin		6 (16.22)		22 (14.77)	0.1183
Nitazoxanide		1 (2.70)		3 (2.01)	0.1172
Ceftriaxone		12 (32.43)		1 (0.67)	**1.68 × 10**^**–12**^
Moxifloxacin/Ciprofloxacin		8 (21.22)		1 (0.67)	**2.60 × 10**^**–8**^
Levofloxacin		16 (43.24)		3 (2.01)	**1.30 × 10**^**–14**^
Ondansetron		4 (10.81)		1 (0.67)	**2.15 × 10**^**–4**^
Enoxaparin		10 (27.03)		1 (0.67)	**2.15 × 10**^**–10**^
Corticoid		15 (40.54)		1 (0.67)	**8.66 × 10**^**–16**^
Comorbidities	**38**		**157**		
Any Comorbidity		27 (71.05)		71 (45.22)	**0.001**
Systemic Arterial Hypertension		19 (50.00)		22 (14.02)	**3.36 × 10**^**–7**^
Asthma		3 (7.89)		22 (14.02)	0.1529
Rhinitis		1 (2.63)		6 (3.82)	0.2415
Hypothyroidism		2 (5.26)		10 (6.37)	0.2493
Obesity		13 (34.21)		3 (1.91)	**1.46 × 10**^**–11**^
Diabetes		10 (26.32)		9 (5.73)	**1.25 × 10**^**–4**^
Hypercholesterolemia		3 (7.89)		1 (0.64)	0.0034
Anxiety/Depression		2 (5.26)		3 (1.91)	0.1210
Chronic Therapy	**28**		**129**		
Any Chronic Therapy		23 (82.14)		61 (47.29)	0.0065
Angiotensin Receptor Blockers		11 (39.29)		11 (08.53)	**1.20 × 10**^**–4**^
ACE Inhibitors		2 (7.14)		3 (2.33)	0.4208
Serotonin Selective Reuptake Inhibitor		2 (7.14)		7 (5.43)	0.9392
β_2_ Adrenergic Agonists		2 (7.14)		6 (4.65)	0.8627
Thiazide Diuretics		7 (25.00)		11 (8.53)	0.0462
Intranasal Corticoids		1 (3.57)		6 (4.65)	0.9690
β Blockers		4 (14.29)		5 (3.88)	0.0996
Benzodiazepines		2 (07.14)		2 (1.55)	0.2349
Calcium Channel Blockers		3 (10.71)		3 (2.33)	0.1106
Statins		4 (14.29)		4 (3.10)	0.0510
Levothyroxine		1 (3.57)		8 (6.29)	0.8631
Metformin		7 (25.00)		9 (6.98)	0.0169

*p-values for Fisher’s Exact test. ** *p-values* for Wilcoxon Signed Rank test. Significance after Bonferroni adjustment for multiple comparisons (α = 0.001) is represented in bold**.**

As expected, comorbidities were more prevalent in cases, with systemic arterial hypertension, obesity and diabetes showing up as major chronic risk factors for supplemental oxygen intervention during SARS-CoV-2 infection in the present cohort. Although chronic use of medications was similarly distributed among cases and controls, angiotensin receptor blockers were found more frequently among cases, suggesting a possible association of this class of drugs with COVID-19 severity (Table 1). As angiotensin receptor blockers are broadly used to control systemic arterial hypertension, we further investigated whether its association with oxygen therapy requirement during COVID-19 could be a consequence of the strong association found for systemic arterial hypertension per se. Logistic regression analysis was then performed on a sub cohort including all participants with hypertension (41 individuals), and no association between angiotensin receptor blockers and respiratory distress was found (Supplementary Table 1).

### *ACE2* and *TMPRSS2* transcription levels

**Figure 1:**
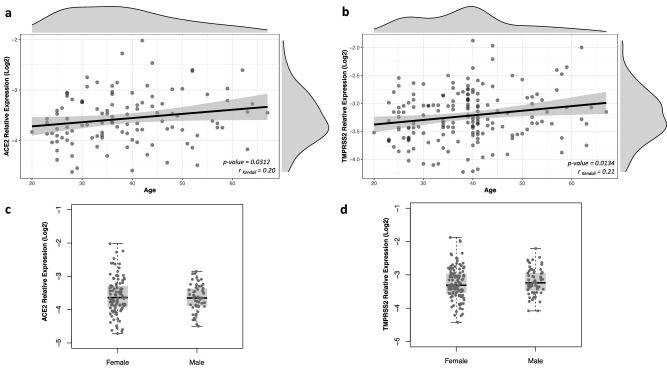
*ACE2* and *TMPRSS2* expression in nasopharyngeal samples from SARS-CoV-2 positive individuals according to age and sex. Relative expression of both genes was determined by RT-qPCR using *B2M* gene as reference and results are shown in log-scale (base 2). The correlation between *ACE2* and *TMPRSS2* expression and age (years) was determined by Kendall rank correlation coefficient and density plots for each variable are depicted externally (**a**,**b**). Boxes represent mean and interquartile range, and whiskers represent upper and lower limit. Linear regression models were applied for comparisons of *ACE2* (**c**) and *TMPRSS2* (**d**) expression between male and female subjects.

Transcription levels were determined in nasopharyngeal samples collected for disease diagnosis and, therefore, all samples were obtained in the course of infection. Expression of both genes was positively correlated to age (r = 0.20; p = 0.03 and r = 0.21; p = 0.01 for *ACE2* and *TMPRSS2*, respectively), while no association with sex was observed (Fig. 1). Expression levels were not associated with any comorbidity or chronic use of medications (Supplementary Fig. 1 and Supplementary Fig. 1).

**Figure 2:**
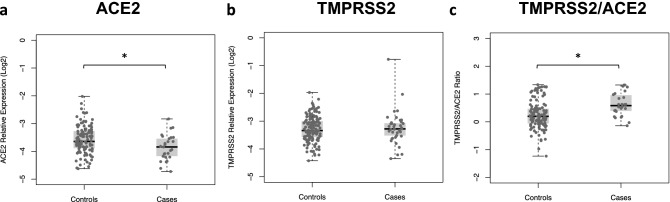
*ACE2* and *TMPRSS2* expression in COVID-19 patients with (cases) and without (controls) respiratory distress. Relative expression of both genes was determined by RT-qPCR using *B2M* gene as reference and results are shown in log-scale (base 2). Boxes represent mean and interquartile range, and whiskers represent upper and lower limit. Linear regression models were applied for comparisons of *ACE2* (**a**) and *TMPRSS2* (**b**) expression levels and *TMPRSS2/ACE2* ratio (**c**) between cases and controls. *p < 0.05.

### Association between *ACE2* and *TMPRSS2* transcription levels and COVID-19 severity

Transcription levels of *ACE2* were significantly lower among cases than in controls (mean values of − 3.80 and − 3.60, respectively), while similar levels of *TMPRSS2* were observed in both groups (Fig. 2a,b). Considering that functional impact of TMPRSS2 on SARS-CoV-2 entry is dependent on the presence of the receptor ACE2, we hypothesize that TMPRSS2 effect could be better explored in relation to *ACE2* expression. TMPRSS2/ACE2 ratios were then determined for each individual and a linear regression analysis showed significantly higher ratios among cases compared to controls (mean values of 0.63 and 0.33, respectively) (Fig. 2c). Next, a stepwise search combined with logistic regression analysis was carried out to define the best covariates to be included in multiple models and then control for confounding. Results showed a better performance for the model including age, systemic arterial hypertension, diabetes and obesity as predictive variables for respiratory distress during COVID-19 (Supplementary Table 2).

**Table 2:** Association between *ACE2* and *TMPRSS2* expression on nasopharynx and respiratory distress during COVID-19.

Relative Log_2_ Expression Level	Cases mean (SD)	Controls mean (SD)	OR (_95%_CI)	_adj_OR (_95%_CI) *
*ACE2*	− 3.85 (0.46)	− 3.60 (0.51)	**0.34** (0.14–0.83)	**0.30** (0.09–0.91)
Total = 172	27	145	p-value = 0.0193	p-value = 0.0318
*TMPRSS2*	− 3.24 (0.61)	− 3.23 (0.46)	0.98 (0.47–2.03)	1.05 (0.43–2.56)
Total = 210	37	173	p-value = 0.9511	p-value = 0.9068
TMPRSS2/ACE2 ratio	0.63 (0.42)	0.33 (0.52)	**3.29** (1.37–7.88)	**4.28** (1.36–13.48)
Total = 171	26	145	p-value = 0.0070	p-value = 0.0131

*OR estimates adjusted for age, systemic arterial hypertension, diabetes and obesity. OR values represent an associated risk/protection according to an increase of 1 log of target’s expression relative to *B2M* gene. SD = standard deviation.

Results of multiple models showed a protective effect for increased expression levels of *ACE2* on nasopharynx either before or after adjustment for covariates (_adj_OR = 0.30; _95%_CI = 0.09–0.91) while no significant association was found for *TMPRSS2* (Table 2). Notably, a strong risk effect of TMPRSS2/ACE2 ratios on COVID-19 severity was found (_adj_OR = 4.28; _95%_CI = 1.36–13.48) (Table 2). Since TMPRSS2/ACE2 fold change is based on transcription levels of both genes and a significant association was already found for *ACE2* alone, a stepwise search combined with regression analyses was performed to compare nested models including both *ACE2* and *TMPRSS2*/*ACE2* ratio along with already selected non-genetic covariates. Results showed that a model including age, systemic arterial hypertension, diabetes, obesity and TMPRSS2/ACE2 minimized the Akaike Information Criteria (AIC = 97.11), suggesting that TMPRSS2/ACE2 ratio outperforms *ACE2* expression predictive power to model COVID-19 respiratory outcome in this cohort (Supplementary Table 3).

### Expression of *ACE2* and *TMPRSS2* genes in lower respiratory tract

**Figure 3:**
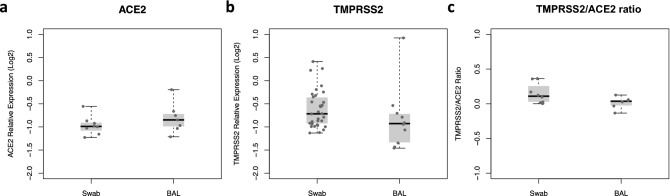
*ACE2* and *TMPRSS2* expression levels on upper and lower respiratory tract of severe COVID-19 patients with respiratory failure. Relative expression levels of *ACE2* (**a**) and *TMPRSS2* (**b**) were determined by RT-qPCR on samples from nasopharyngeal swabs and bronchoalveolar lavages (BAL). TMPRSS2/ACE2 ratio were calculated for each individual and compared between swabs and BAL samples (**c**). Results are represented in log-scale (base 2) relative to *B2M* expression. Boxes represent mean and interquartile range, and whiskers represent upper and lower limit. Linear regression models were applied for comparisons between groups.

To further investigate whether results obtained from the nasopharynx might reflect transcription levels at lower respiratory tract from critically ill COVID-19 individuals, additional 34 nasopharyngeal swabs and 11 bronchoalveolar lavage (BAL) samples were obtained from an independent case-only cohort of individuals. Distribution of age and gender were similar between swab and BAL groups (Supplementary Table 4). Results of linear regression models showed similar transcription levels of *ACE2* and *TMPRSS2* in nasopharyngeal and BAL samples even after adjustment for the same covariates selected after stepwise regression (Fig. 3). No significant differences were found for TMPRSS2/ACE2 ratio whatsoever.

## Discussion

Population-based studies on COVID-19 patients have been elucidating epidemiological factors contributing to disease severity such as age and chronic diseases (systemic arterial hypertension, diabetes and, obesity)^2,5^. Consistent with previous literature findings, age showed up as a major risk factor for the need of oxygen support during SARS-CoV-2 infection in the present study. Comorbidities such as systemic arterial hypertension, diabetes and obesity also appeared here as significant factors associated with respiratory distress.

Prevalence of SARS-CoV-2 induced headache and anosmia has been found to vary considerably between cohorts^17^. Here, 61.5% of total participants reported headache and 57.28% showed olfactory impairment/dysfunction with a remarkably overrepresentation of these outcomes among controls. No significant differences were found for general chronic therapies between cases and controls. However, previous in silico findings warned for potentially higher *ACE2* or *TMPRSS2* levels during systemic arterial hypertension treatments^18^. Although we did not find any evidence of chronic therapy influencing *ACE2* or *TMPRSS2* expression (Supplementary Fig. 2), angiotensin receptor blockers were significantly more frequent among cases, suggesting an impact on COVID-19 severity. After a sub cohort analysis, the association was found to be driven by systemic arterial hypertension per se (Supplementary Table 1). Although preliminary, this supports recent findings that the use of this class of drugs is not associated with disease severity during SARS-CoV-2 infections^19–21^.

ACE2 has been described as the entry receptor for SARS-CoV-2 infection and TMPRSS2 as a major priming enzyme relevant to its fusion on the cell membrane^6^. Since the beginning of the pandemic, a lot of questions rely on how expression of these host factors could impact on COVID-19 pathogenesis. *ACE2* and *TMPRSS2* levels have previously been found lower in children than in young adults’ upper airways, raising the hypothesis that they could reflect age-related susceptibility to COVID-19^8,22^. However, expression levels of these genes were not yet assessed in different age groups during infection. Here, in addition to the association with severity, age also appears to impact *ACE2* and *TMPRSS2* expression levels at the nasopharyngeal epithelium of SARS-CoV-2 infected individuals.

In the present study, we characterized the transcription levels of both genes in nasopharyngeal samples of COVID-19 patients and investigated their association with respiratory distress using a case–control study design. After logistic regression analysis, higher nasopharyngeal expression of *ACE2* was significantly associated with protection against respiratory distress requiring supplemental oxygen during COVID-19. *ACE2* protective effect may be supported by recent evidence of its “interferon stimulated gene” attributes, where higher levels of *ACE2* transcripts could be a consequence of higher interferon activity induced by viral replication itself^23^ and then possibly acting as a hallmark of a more robust antiviral response^7^. Experimental data showing *ACE2* upregulation on smokers suggests a risk effect for IFN induced overexpression of this gene in the lungs by IFN signaling activation^11,22,24^. However, contrasting with the pattern expected for the upper respiratory tract, exacerbation of IFN signaling in lungs could worsen coronavirus associated SARS by increasing inflammation and induce intense endothelial/alveolar damage^25–27^.

A risk effect for *TMPRSS2* levels on COVID-19 severity could be expected once this enzyme triggers the viral S protein priming after its recognition by ACE2 entry receptor and is considered an important player host susceptibility to SARS-CoV-2^6^. Moreover, *TMPRSS2* expression was also found to be reshaped by smoking habits, a known risk factor for SARS^22^. However, since its functional activity on viral fusion depends on ACE2 mediated viral adsorption, an association could only be seen in this study when both targets were evaluated together. In fact, higher *TMPRSS2/ACE2* ratios showed a prominent risk effect for respiratory distress requiring oxygen therapy during COVID-19 and comparisons between multiple models revealed that *TMPRSS2/ACE2* ratio can be even more informative to model disease severity than *ACE2* expression alone. These data suggest that further expression studies on SARS-CoV-2 pathogenesis should consider evaluating co-expression of both genes.

Finally, expression analysis from nasopharyngeal swab samples compared to BAL on an independent case-only cohort demonstrates similar expression patterns of *ACE2*, *TMPRSS2* and *TMPRSS2/ACE2* on upper and lower respiratory tracts of COVID-19 patients under mechanical ventilation. Recent work has shown that *ACE2* and *TMPRSS2* expression are significantly higher in nasal than in bronchial tissues in children and adults^22^. Our data suggests that, after viral colonization of both upper and lower respiratory tracts, *ACE2* and *TMPRSS2* assessed by nasopharyngeal swabs seems to reflect what is found on BAL samples. Then, expression levels of these genes could be further explored as potential biomarkers.

An important limitation of this study is that our data does not allow a clear interpretation of *ACE2* and *TMPRSS2* levels as predictors of disease severity, since case samples were obtained at the moment of hospital admission to initiate oxygen therapy. Prospective studies should then be carried out to better explore the prediction effect of these factors on COVID-19 severity. We also would like to address that, due to sample size limitations in the case group, we were unable to perform stratification analysis for different methods of oxygen therapy (nasal cannula, noninvasive mechanical ventilation and intubation). Then, we encourage future studies with greater sample sizes to do so in order to better define the contribution of *ACE2* and *TMPRRS2* expression levels on respiratory outcome severity.

Taken together, the present study shows an association between nasopharyngeal expression of *ACE2* and *TMPRSS2* genes and the need of oxygen therapy during COVID-19. Our data also supports that *TMPRSS2* effect on modelling disease severity may be dependent on *ACE2* levels, suggesting that the impact of this serine protease on COVID-19 might be better explored in combination to its partner *ACE2*.

## Supplementary Information

Supplementary Information
