# Supplementary material for: Association between ACE2 and TMPRSS2 nasopharyngeal expression and COVID-19 respiratory distress
Source: Sci Rep. 2021 May 6;11:9658. doi: 10.1038/s41598-021-88944-8 (PMC8102547; doi:10.1038/s41598-021-88944-8)
Supplement: Supplementary file 1 — Supplementary Information [file 41598_2021_88944_MOESM1_ESM.pdf]

Association between *ACE2* and *TMPRSS2* Nasopharyngeal Expression and COVID-19 respiratory distress

Átila Duque Rossi[1], João Locke Ferreira de Araújo[2], Tailah Bernardo de Almeida[3], Marcelo Ribeiro-Alves[4], Camila de Almeida Velozo[1], Jéssica Maciel[1], Isabela de Carvalho Leitão[5], Sâmila Natiane Ferreira[6], Jéssica da Silva Oliveira[6], Hugo José Alves[2], Helena Toledo Scheid[7], Débora Souza Faffe[5], Rafael Mello Galliez[7], Renata Eliane de Ávila[8], Gustavo Gomes Resende [9], Mauro Martins Teixeira[10], COVID-19 UFRJ Workgroup, Orlando Costa Ferreira[1], Terezinha Marta P. P. Castiñeiras[7], Renan Pedra Souza[2], Amilcar Tanuri[1], Renato Santana Aguiar[2], Shana Priscila Coutinho Barroso[6], Cynthia Chester Cardoso[1]*

[1]Laboratório de Virologia Molecular, Departamento de Genética, Instituto de Biologia, Universidade Federal do Rio de Janeiro, Rio de Janeiro, Postal Code: 21941-902, Brazil.

[2]Laboratório de Biologia Integrativa, Departamento de Genética, Ecologia e Evolução, Instituto de Ciências Biológicas, Universidade Federal de Minas Gerais, Belo Horizonte, Postal Code: 31270-901, Brazil.

[3]Instituto de Estudos do Mar Almirante Paulo Moreira, Marinha do Brasil, Arraial do Cabo, Postal Code: 28930-000,

[4]Instituto Nacional de Infectologia Evandro Chagas, Fiocruz, Rio de Janeiro, Postal Code: 21040-360, Brazil.

[5]Instituto de Biofísica, Universidade Federal do Rio de Janeiro, Rio de Janeiro, Postal Code: 21941-902, Brazil.

[6]Instituto de Pesquisas Biomédicas, Hospital Naval Marcílio Dias, Marinha do Brasil, Rio de Janeiro, Postal Code: 20725-090, Brazil.

[7]Faculdade de Medicina, Universidade Federal do Rio de Janeiro, Rio de Janeiro, Postal Code: 21941-902, Brazil.

[8]Hospital Eduardo de Menezes, Belo Horizonte, Postal Code: 30622-020, Brazil.
[9]Hospital das Clínicas, Universidade Federal de Minas Gerais (HC-UFMG/EBSERH), Belo Horizonte, Postal Code: 30130-100, Brazil.
[10]Departamento de Bioquímica e Imunologia, Instituto de Ciências Biológicas, Universidade Federal de Minas Gerais, Belo Horizonte, Postal Code: 31270-901, Brazil.
[11]Centro de Triagem e Diagnóstico COVID-19 UFRJ, Universidade Federal do Rio de Janeiro, Rio de Janeiro, Postal Code: 21941-902, Brazil.

***Corresponding Author**
Cynthia Chester Cardoso
Laboratório de Virologia Molecular, Departamento de Genética, Instituto de Biologia, Universidade Federal do Rio de Janeiro
Av. Carlos Chagas Filho 373, CCS, Bloco A, Sala 121 Ilha do Fundão, RJ, 21941-902, Brazil
Phone number: 55-21-25643353
email: cynthiac@biologia.ufrj.br

Supplementary Table 1: Association between chronic use of angiotensin receptor blockers and COVID-19 respiratory distress.

**Total Cohort (N = 213)**				
	Cases (%)	Controls (%)	OR ($_{95\%}$CI)	*p-value*
Chronic Angiotensin Receptor Blockers Use	38 (17.84)	175 (82.16)	6.94 (2.61 – 18.46)	**2.07 x 10^{-4}**
**Subset of Systemic Arterial Hypertension Individuals (N = 41)**				
	Cases (%)	Controls (%)	OR ($_{95\%}$CI)	*p-value*
Chronic Angiotensin Receptor Blockers Use	19 (46.34)	22 (53.66)	3.24 (0.65 – 19.09)	0.1672

Supplementary Table 2: Stepwise regression analysis for selection of covariates associated to COVID-19 respiratory distress.

Explanatory Variables	Deviance	AIC
**Age + Systemic Arterial Hypertension + Diabetes + Obesity**	**122.52**	**130.51**
- Systemic Arterial Hypertension	121.57	131.57
- Diabetes	125.80	131.80
+ Any Comorbidity	122.03	132.03
+ Sex	122.25	132.25
- Obesity	141.76	147.76
- Age	145.47	151.47

AIC = Akaike Information Criteria. Plus and minus symbols indicates whether the variable was removed (-) or included (+) in the model.

Supplementary Table 3: Stepwise regression analysis for variables associated with COVID-19 respiratory distress including *ACE2* and *TMPRSS2* expression levels.

Explanatory Variables	Deviance	AIC
**Age + Systemic Arterial Hypertension + Diabetes + Obesity + TMPRSS2/ACE2**	**85.11**	**97.11**
- Systemic Arterial Hypertension	87.21	97.21
+ ACE2	84.17	98.17
+ Any Comorbidity	84.83	98.83
+ Sex	84.99	98.99
- Diabetes	89.19	99.19
- TMPRSS2/ACE2	91.85	101.85
- Age	96.84	106.84
- Obesity	99.07	109.07

AIC = Akaike Information Criteria. Plus and minus symbols indicates whether the variable was removed (-) or included (+) in the model.

Supplementary Table 4: Characteristics of patients included in comparisons between nasopharyngeal swab and bronchoalveolar lavage samples.

	Group	
	BAL (N=11)	SWAB (N=34)
**Age***	62 (±7)	57 (±14)
**Sex**		
Male	6 (54·5%)	19 (55·9%)
Female	5 (45·5%)	15 (44·1%)
**Comorbidity**		
Systemic Arterial Hypertension	5 (55·5%)	20 (58·8%)
Obesity	3 (27·3%)	11 (32·3%)
Diabetes	5 (55·5%)	9 (26·5%)

*data represented as mean ± standard deviation. BAL = bronchoalveolar lavage fluid; swab = nasopharyngeal swab samples.

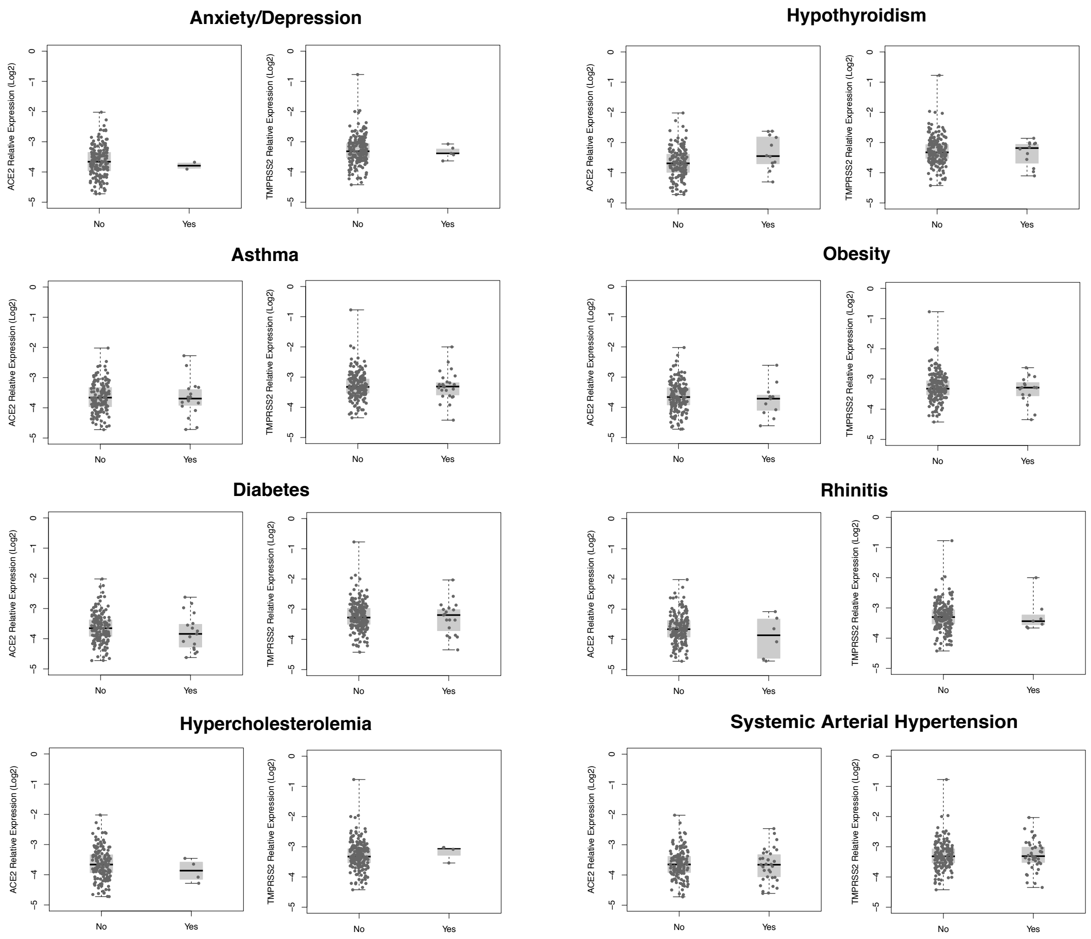

Supplementary Figure 1: Nasopharyngeal expression of *ACE2* and *TMPRSS2* in individuals with mild COVID-19 according to different chronic diseases. Expression levels were assessed by RT-qPCR and are shown in log-scale (base 2) taking *B2M* expression as reference. Boxes represent mean and interquartile range, and whiskers stand for upper and lower limits. Comparisons between groups were made by linear regression models.

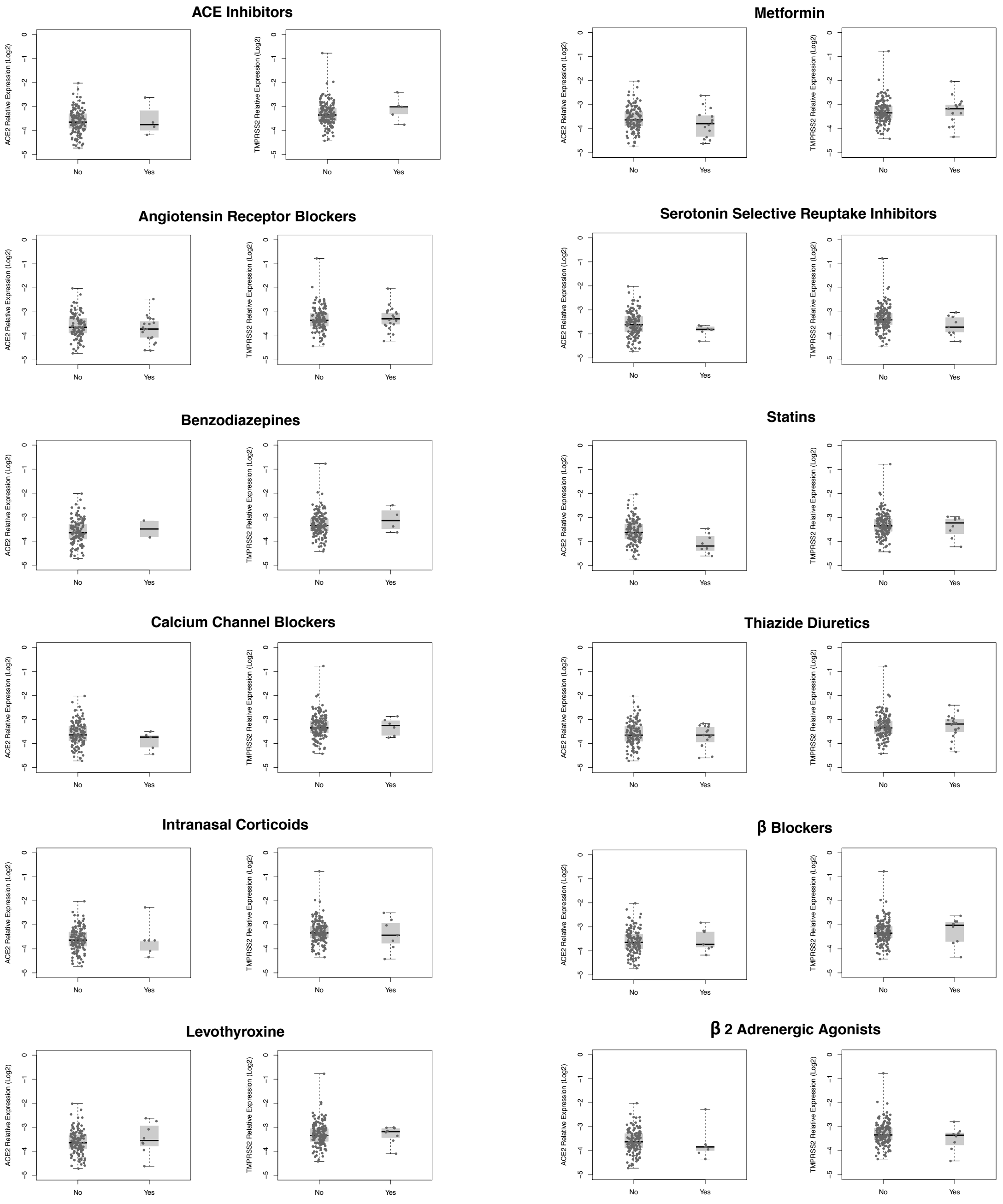

Supplementary Figure 2: Nasopharyngeal expression of *ACE2* and *TMPRSS2* in individuals with mild COVID-19 according to chronic therapy use. Expression levels were assessed by RT-qPCR plotted in log-scale (base 2) relative to *B2M* expression. Boxes represent mean and interquartile range, and whiskers stand for upper and lower limits. Comparisons between groups were made by linear regression models.
